# Racism and health service utilisation: A systematic review and meta-analysis

**DOI:** 10.1371/journal.pone.0189900

**Published:** 2017-12-18

**Authors:** Jehonathan Ben, Donna Cormack, Ricci Harris, Yin Paradies

**Affiliations:** 1 Alfred Deakin Institute for Citizenship and Globalization, Faculty of Arts and Education, Deakin University, Melbourne, Victoria, Australia; 2 Eru Pōmare Māori Health Research Centre, Department of Public Health, University of Otago, Wellington South, New Zealand; Leibniz Institute for Prvention Research and Epidemiology BIPS, GERMANY

## Abstract

Although racism has been posited as driver of racial/ethnic inequities in healthcare, the relationship between racism and health service use and experience has yet to be systematically reviewed or meta-analysed. This paper presents a systematic review and meta-analysis of quantitative empirical studies that report associations between self-reported racism and various measures of healthcare service utilisation. Data were reviewed and extracted from 83 papers reporting 70 studies. Studies included 250,850 participants and were conducted predominately in the U.S. The meta-analysis included 59 papers reporting 52 studies, which were analysed using random effects models and mean weighted effect sizes. Racism was associated with more negative patient experiences of health services (HSU-E) (OR = 0.351 (95% CI [0.236,0.521], k = 19), including lower levels of healthcare-related trust, satisfaction, and communication. Racism was not associated with health service use (HSU-U) as an outcome group, and was not associated with most individual HSU-U outcomes, including having had examinations, health service visits and admissions to health professionals and services. Racism was associated with health service use outcomes such as delaying/not getting healthcare, and lack of adherence to treatment uptake, although these effects may be influenced by a small sample of studies, and publication bias, respectively. Limitations to the literature reviewed in terms of study designs, sampling methods and measurements are discussed along with suggested future directions in the field.

## Introduction

Differential and inequitable patterns of healthcare access, utilisation and quality by race/ethnicity are evident in many countries and for a range of healthcare indicators [1–6]. Racial/ethnic inequities in patient experience, including levels of satisfaction and trust, have also been demonstrated [7–10]. While racism has been posited as driver of racial/ethnic inequities in healthcare [5], the existing studies focused on how racial discrimination affects use and experience of health services have yet to be systematically reviewed or meta-analysed [11, 12].

Racism is a complex social system underpinned by unequal power relations and beliefs about ‘race’ and related systems of categorisation, expressed through attitudes, prejudice, and discrimination as well as racialised practices and structures [13]. Research on the health impacts of racism and its role in (re)producing racial/ethnic inequities in health through entrenched systems of privilege and oppression has increased substantially in recent years. The negative impacts of racism on physical and mental health and on exposure to health-damaging factors is well-documented, with the bulk of this research focused on measures of self-reported personal experiences of racism [14, 15].

Although less interrogated, racism may also impact the quality of healthcare, how individuals access and use health services, and experiences and perceptions of healthcare, with flow-on effects for health and racial/ethnic inequities [16]. Racial discrimination experienced within the healthcare setting, or in society more generally, may influence how people perceive the healthcare system, how they engage with health services and providers, and the patterns and quality of their healthcare access [16, 17]. Experiences of racism have the potential to impact on patient satisfaction, levels of trust, and perceived quality of healthcare interactions, and to influence whether individuals follow provider recommendations as well as impacting on their future patterns of health service use [17–19]. Reported racism may be directly linked to healthcare-related outcomes in that it may capture discriminatory treatment experienced by individuals in their interactions with healthcare providers [12]. This may operate without physicians’ awareness as is demonstrated by increasing evidence that physician racial/ethnic bias can affect the quality of communication with patients and patient management decisions [20, 21]. Stereotype threat among patients, influenced by past experiences of racism, may influence patients’ behaviour in subtle ways and can also increase physicians’ racial/ethnic bias through the reinforcement of racial/ethnic stereotypes [16]. After experiencing racism in healthcare or other social settings, individuals may seek less contact with the healthcare system. More indirectly, experiences of racism may limit the time and energy required to access health care [22]. Conversely, racism may be indirectly associated with an increased need for healthcare, due to its negative impacts on physical and mental health [23].

Drawing on approaches used in recent reviews of self-reported racism and health [14, 24], this paper describes a systematic review and meta-analysis of quantitative empirical literature that reports associations between self-reported racism and healthcare-related measures. Although publication of primary studies in this area has seen considerable growth in recent years, this empirical evidence base has yet to be synthesised via a systematic review and meta-analysis. Consequently, the overall magnitude and direction of the relationship between racism and healthcare service utilisation (HSU) outcomes are not yet known. This paper aims to address these gaps and provide a comprehensive, up-to-date overview of this burgeoning field.

## Methods

### Search strategy

The literature search for this review focused on papers reporting direct associations between racism and HSU outcomes. Two separate searches were conducted in English, and papers in languages other than English were excluded. The first, principal search, covered four databases, namely CINAHL, MEDLINE, PsychINFO, and PubMed. The search originally ran until September 2011, and was extended until the end of October 2015 (earliest date limit was not specified). A second, supplementary, search entailed re-screening search results from a recent systematic review and meta-analysis conducted by two of the authors [14] on racism and health (which included HSU outcomes as search terms). The supplementary search covered the databases Academic Search Premier, CINAHL, ERIC, MEDLINE, ProQuest (for dissertations/theses), PsychINFO, Sociological Abstracts, Social Work Abstracts and Web of Science. It originally ran until October 2013 and was extended until October 2015 (earliest date limit was not specified). The supplementary search aimed to broaden the principal search, through covering additional databases and by including additional search terms. For a list of search terms used in each search, see S1 Appendix. The searches overlapped to a certain extent. The results of the principal search were screened first. Duplicates that were found also by the supplementary search were removed, and the supplementary search was used to identify additional papers that were not previously located. Each search was also supplemented by searching bibliographies of included empirical papers and review articles. Although the searches originally included both published and unpublished materials, only published papers were included in the current review. Theses, dissertations, and conference papers and presentations were excluded during screening.

### Inclusion criteria

To be included in the review, papers had to report empirical research and contain quantitative data on the association between racism and HSU outcomes.

#### Exposure

This review uses reported racism as an exposure. Exposure measures included in this review are racism, discrimination, prejudice, stereotypes, maltreatment, aggression and related terms, where possible reasons for these include race, skin colour, ethnicity, religion, and language. This review includes two types of racism: 1) self-reported racism, experienced directly in interpersonal contact; and 2) self reported racism experienced indirectly, when it is directed towards a group which the person is a member of, for example based on skin colour, race or ethnicity. Other types of exposure to reported racism (e.g., vicarious experiences of witnessing racism, proxy reports, internalized racism) were originally included, but all studies reporting them were excluded on other grounds.

General measures of discrimination, where the specific effect of racism on HSU outcomes cannot be isolated, were excluded, unless the measure was modified to explicitly specify race, ethnicity, skin colour, etc. as the reason/s for discrimination. When the majority of items within an exposure measure assessed racism while all remaining items assessed discrimination broadly defined (without specifying the reason for discrimination), the measure was included. Measures of exposure to discrimination due to other reasons, such as gender, sexuality, socioeconomic status and so on, were excluded. Exposure measures that combine racism with responses to racism or with possible health outcomes were excluded to allow for assessment of the association between exposure and outcome measures as two separate constructs, and to avoid possible conflation of racism with potentially related outcomes. We therefore excluded exposures to race-related stress, discrimination-distress, and other exposures combining racism with responses to racism (e.g., how much respondents are bothered by racism) or relating racism to health within the same instrument. Finally, because this study focuses on observational studies of racism as reported by research participants, we excluded measures of racism that were ecological (e.g., racial segregation), experimental (e.g., videos, vignettes, tasks) as well as other exposures where racism was assessed by the researcher.

#### Outcomes

This review includes two types of HSU outcomes: outcomes relating to patients’ use of healthcare (including access), and outcomes relating to patients’ experiences. This distinguishes between measures of contact or engagement with healthcare (e.g. access, uptake) and patient perceptions of their healthcare experience, which captures aspects of quality of care. Patients’ experiences relating to HSU (HSU-E) include the following outcomes (in brackets the abbreviations of outcome names as used throughout this review): 1) communication and relationships with health service professionals (COM); 2) satisfaction with health services and perceived quality of care (SAT); 3) trust in healthcare systems and professionals (TRUST); and 4) a mix of different HSU-E outcomes (HSU-EMIX).

Outcomes relating to Health Service Use (HSU-U) include the following: 5) having had examinations, tests, screening and/or checks (EXAM); 6) having followed prescriptions, recommended treatments and behaviours and having taken medications and vaccinations (UPTAKE); 7) visits to health professionals (VP); 8) visits and admissions to hospitals and emergency departments (VH); 9) delaying/not getting healthcare (self-assessed) (DELAY); 10) having insurance and/or healthcare (INS); and 11) a mix of different HSU-U outcomes (HSU-UMIX).

Several HSU-related outcomes were excluded because they were reported by very few papers and/or were conceptually different from the outcome groups listed above. Examples of outcomes that were excluded for being too rarely reported include: length of patient-provider relationship, provider warmth/respect, whether care was cost-prohibitive, and attitudes and intentions to use health services. Examples of outcomes that were excluded for conceptual reasons include: source of healthcare (e.g., source type, usual source, use of informal services), knowledge, information, and type and quality of information about health services.

In several cases, papers included relevant exposure and outcome measures, yet they were excluded because they did not examine or did not report an association between them. Associations were also excluded when racism was treated as an outcome in multivariate analyses (rather than as an exposure).

### Screening

Online search results were imported into Endnote X7 [25], and duplicates were deleted. The online search yielded 3,666 search results, while supplementary searches of the wider literature on racism and health initially yielded over 28,000 search results.

First, one reviewer screened the titles and abstracts of all papers to assess their eligibility for inclusion, i.e. whether titles and abstracts indicated papers may report empirical research and contain quantitative data on the association between racism and one or more HSU outcomes. In addition, a sample of titles and abstracts were double screened. Disagreements between the reviewers were resolved by consensus. At the end of screening titles and abstracts, 319 references were retained: 127 were found through the principal search, and 192 were found through the supplementary search.

**Fig 1:**
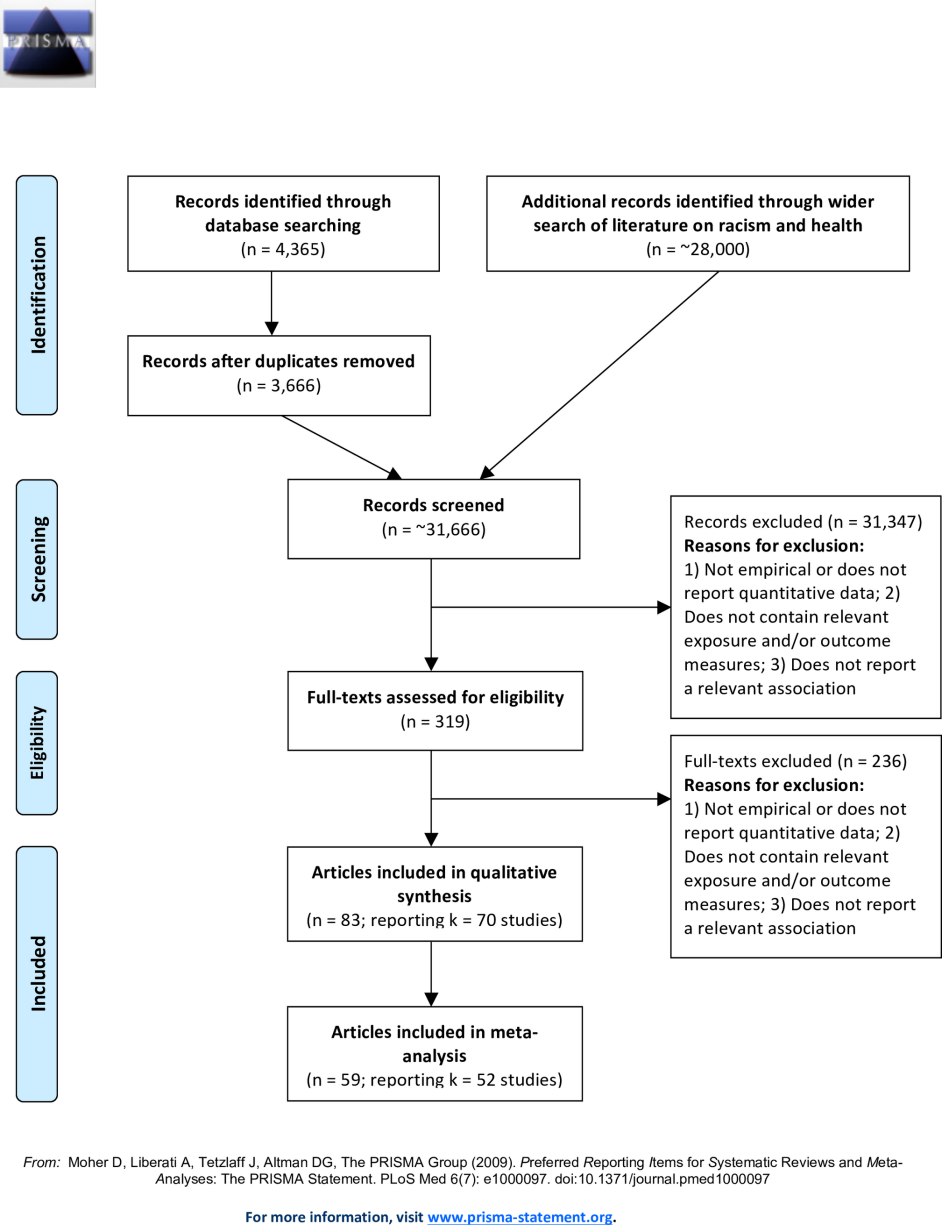
PRISMA flow diagram.

The full-text of each reference was then reviewed for its inclusion eligibility by two independent reviewers. Disagreements were resolved by consensus between the reviewers. The main reasons for exclusion were papers reporting irrelevant outcomes and/or irrelevant exposures. At the end of screening full-texts, 57 papers were retained in the principal search, and an additional 26 papers were retained in the supplementary search. The final sample size of papers included in this review consists of 83 papers [18, 19, 23, 26–105]. Fig 1 summarises the numbers of papers at each stage of screening.

### Data extraction and coding

Data from each paper were extracted and reviewed by two reviewers. One reviewer (DC, JB or MVT) extracted data into a Microsoft Excel spreadsheet and another reviewer (RH or YP) reviewed the data that were extracted. Five types of data were extracted from each paper, namely at the level of the study, participants, exposure measures, outcome measures, and effect size data. Disagreements were discussed and resolved by consensus between the reviewers. Data coding was undertaken by one reviewer, and coding decisions were made in discussion with a second reviewer.

This review includes papers that contain unadjusted bivariate associations between racism and HSU as well as associations between racism and HSU that adjust for different covariates. While the meta-analysis focuses exclusively on unadjusted associations between racism and HSU (see details below), the review also includes papers that only report associations that adjust for covariates. These papers are included in the descriptive analysis, and some of them are also included in the vote-counting analysis of associations that adjust for age, gender and race.

### Data integration and analysis

Odds ratios (ORs) and 95% Confidence Intervals (CIs) were the most commonly used metrics for measuring associations between racism and HSU in the papers reviewed, and are employed as the measures of effect size in the meta-analysis aspect of this study. Other metrics were converted to ORs and 95% CIs where possible. One reviewer examined whether papers reported appropriate and sufficient data to be included in the meta-analysis, and decisions regarding exclusion were discussed with a second reviewer.

The following data formats were converted to ORs using Comprehensive Meta Analysis (CMA) Version 2.2 [106]: 1) correlation coefficient and sample size; 2) cross-tabulation (2x2) of events and non-events (racism/no racism x poor/good health); 3) means, standard deviations and sample sizes for two groups (racism and no racism); 4) means and samples sizes for two groups (racism and no racism), and an independent group p-value; and 5) p -value and sample size for correlation coefficient. In some papers, a p-level was reported rather than the exact p-value. Where the association was significant and only the p-level was reported, the p-value was conservatively recorded just below the p-level used in the study (e.g., a significant p-value at <0.0001 was recorded as p = 0.00009999). Where the p-value was not significant and its exact value not reported, the association was conservatively recorded as zero (see [107] for a similar approach). Altogether, such conservative approximations of p-values and effect sizes were used to retain 34 associations, accounting for 17.8% of the 191 associations used in the meta-analysis.

Several papers did not report appropriate and/or sufficient data to be converted into ORs and 95% CIs, and were included in this review, but excluded from the meta-analysis. Adjusted analyses of racism and HSU outcomes, which commonly contain different sets of covariates, were excluded from the meta-analysis as well since combining adjusted data with unadjusted data in meta-analysis limits transformations between different effect sizes and poses difficulties in interpreting the effects of covariates ([24] pg 534, [108]).

Effect size data were coded using CMA. In the analysis, an OR lower than 1 indicates that an increase in racism is associated with decreased HSU, i.e. an inverse association between racism and HSU. Where a paper coded ORs and CIs in the opposite way, i.e., where an OR lower than 1 indicates that an increase in racism is associated with increased HSU, ORs and 95% CIs were reverse-coded using 1/OR.

Using CMA, weighted effect sizes were calculated to account for variation in sample sizes, thus giving more weight to effect sizes from larger samples. When a study comprised multiple associations between racism and HSU for the same participant group/s, to ensure associations were independent, averaging was used across these associations. All relevant associations were extracted and a shifting unit of analysis approach (e.g., [109]) was employed to conduct analyses both at the level of individual outcomes (e.g., for the outcome ‘visits to health professionals’ (VP) only), and at the level of broader outcome groups (e.g., for the outcome group HSU-U, consisting of the outcome VP among other outcomes), in which case we averaged associations for different outcomes relating to health service use, including outcomes such as visits to health professionals and visits and admissions to hospitals and emergency departments.

The review included 5 studies that were each reported in two papers or more. These studies were reported in 15 papers altogether (*k* indicating the number of papers reporting each study): Behavioral Risk Factor Surveillance System (BRFSS) (k = 6), California Health Interview Survey (CHIS) (k = 3), Latin American Cancer Research Coalition (LACRC) (k = 2), National Latino and Asian American Study (NLAAS) (k = 2), Sinai Improving Community Health Survey (k = 2). In addition, we examined associations from papers by the same first author/s where the names of studies or data sources were not mentioned but where the methodology and sample characteristics were identical or nearly identical, suggesting the same data may have been used in multiple papers. Three such potential data sources, each reported in two papers, were identified in discussion between two reviewers. Associations from the same study that were reported in multiple papers were averaged using CMA. The 83 papers included in this review report data from 70 studies, while the meta-analysis consists of 59 papers that report data from 52 studies.

Similar to previous meta-analyses on racism/discrimination and health [14, 24], a random effects model was used for aggregating effect sizes in all analyses. Previous studies on racism and health have viewed this model as more appropriate than a fixed effects model because of the various differences across studies, including methods, instrumentation and sample characteristics (e.g., [24]), and given that the aim of this study is to generalise findings to the population of studies on racism and HSU outcomes (see also [110, 111]). Mixed effect models were used in moderation analyses, as a more conservative approach that enables testing of differences between different moderator levels (e.g., [111]).

#### Moderation analyses

The direction and strength of the relationship between a predictor variable and an outcome variable may be influenced by variables known as moderators [112]. Existing health or medical conditions and the setting where racism was experienced (healthcare focused/not healthcare focused) were used as moderators.

Co-morbidities (i.e. health/medical conditions) are drivers of both the need for services from the healthcare system and, due to increased contact with service providers, possible exposure to racism in healthcare settings. Additionally, health/medical conditions could be one of the pathways by which experience of racism is associated with healthcare outcomes, i.e. racism may affect health, which may affect HSU. Such potentially complex interactions may result in a different relationship between racism and HSU at various levels of co-morbidity. In terms of racism setting, there is initial evidence that racism experienced in healthcare vs. other settings may differ in their strength of association [57, 58, 113].

Moderation analyses were run per outcome group, where at least two levels of the moderator each included 5 or more studies (for a similar approach see [14]). Study was the unit of analysis in all moderation analyses.

#### Vote-counting analysis

To supplement our meta-analysis of unadjusted associations between racism and HSU outcomes, the findings of studies adjusting for age, gender, and race were summarised using vote-counting methods. These variables were selected as those which were most commonly reported across studies of racism and HSU. Vote-counting included analyses that adjust for age among participants over 18 years, for gender, and for at least two racial groups (African American and White American). In most cases, studies also adjusted for additional covariates. Altogether, there were 9 papers (reporting data from 7 studies) with empirical data on the association between racism and HSU outcomes that adjust for these covariates.

#### Publication bias assessment

We used three methods to assess publication bias: 1) Funnel plots were produced, and their symmetry was examined to visually assess evidence of bias; 2) Egger’s weighted regression method [114] was employed, and the regression intercept’s significance was examined for statistical evidence of bias; 3) A failsafe N was calculated to assess whether the effect may be a result of publication bias due to the “file-drawer problem” where significant results may be more likely to get published than non-significant results that remain in the researcher’s ‘file drawer’. Failsafe N estimates the number of additional, unlocated studies with an average effect size of zero that would be required to change a significant result to a non-significant one [115]. We used Rosenthal’s criterion that the failsafe N value should be at least 5*k* + 10 the number of studies included in the meta-analysis [116]. The trim-and-fill method [117, 118] was used to adjust for missing (un-reported) studies, to estimate what the effect size would be in the absence of bias. All publication bias tests, as well as trim-and-fill point estimates, were calculated using CMA.

## Results

### Descriptive analysis

The results of the descriptive analysis are presented in Table 1. This analysis reports on data per paper rather than per study or data source, which is in line with previous reviews and meta-analyses. Since multiple papers reporting the same study often examine different subsets of the data, reporting descriptive data at the level of the study/data source was not feasible. This approach potentially double counts participants from 8 studies, each reported in multiple papers (altogether 21 papers), which we recognize as a potentially minor bias.

**Table 1:** Descriptive analysis.

Variable	Groups	# of papers reporting	% of papers reporting
Total number of papers		83	100%
Type of publication	Academic journal	82	98.8%
	Report	1	1.2%
Year of publication	1997–2000	2	2.4%
	2001–2005	10	12.0%
	2006–2010	30	36.1%
	2011-October 2015	41	49.4%
Country of research	United States	79	95.2%
	Australia	1	1.2%
	The Netherlands	1	1.2%
	New Zealand	1	1.2%
	Sweden	1	1.2%
Sampling procedure	Non-representative	43	51.8%
	Representative	39	47.0%
	Not reported	1	1.2%
Data type	Cross-sectional	80	96.4%
	Longitudinal	3	3.6%
Recruitment from healthcare settings	Yes	32	38.6%
	No	48	57.8%
	Both	3	3.6%
Sample size*	0–100	9	10.8%
* *	101–200	13	15.7%
* *	201–300	10	12.0%
* *	301–1000	16	19.3%
* *	1001+	35	42.2%
Age	Children and adolescents (under 18)	2	2.4%
	Adults (18 and older)	71	85.5%
	Mixed age groups	2	2.4%
	Not reported	8	9.6%
Sex	Female only	15	18.1%
	Male only	7	8.4%
	Male and female	61	73.5%
Racial/ethnic group**	African/Black American	64	77.1%
	Hispanic/Latino/a American	34	41.0%
	Asian American	18	21.7%
	European/White American	40	48.2%
	Native American	7	8.4%
	Non-American native/indigenous	2	2.4%
Specific health/medical condition	Diabetes	10	12.0%
	HIV	6	7.2%
	Heart conditions (myocardial infarction; cardiac patients)	2	2.4%
	Hypertension	2	2.4%
	Osteoarthritis	2	2.4%
	Other (1 paper per condition): cancer, mental health disorders, Systemic lupus, erythematosus (SEL)	3	3.6%
	Samples not focused on patients with specific conditions/not reported	58	69.9%
Exposure instrument name**	Everyday Discrimination Scale (EDS) ***	13	15.7%
	Experiences of Discrimination (EOD)	12	14.5%
	Racism in Health Care Index	7	8.4%
	Discrimination in Medical Settings (DMS)	5	6.0%
	Schedule of Racist Events (SRE)	3	3.6%
	Racism and life experience scales (RaLES)	3	3.6%
	Multiple Discrimination Scale (MDS)	3	3.6%
	Perceptions of Racism Scale (PRS; Green, 1995)	2	2.4%
Exposure number of items**	Single item/s	33	39.8%
	2–8 items	33	39.8%
	9 or more	19	22.9%
	Not reported	1	1.2%
Exposure focus on healthcare**	Healthcare focused	49	59.0%
	Not healthcare focused	40	48.2%
	Not reported	1	1.2%
Exposure type: direct/indirect**	Direct	69	83.1%
	Indirect	14	16.9%
	Mixed (instruments including subscales from both levels)	4	4.8%
	Not reported	1	1.2%
Administration of exposure**	Other-administered	59	71.1%
	Self-administered	19	22.9%
	Not reported	5	6.0%
Timeframe of exposure**	Last 12 months	17	20.5%
	Last 2 years/last 5 years	2	2.4%
	Lifetime	27	32.5%
	Not reported/not specified (includes 'everyday')	46	55.4%
	Mixed (instruments including subscales from both levels)	3	3.6%
Outcomes–HSU-E**	SAT	15	18.1%
	TRUST	12	14.5%
	COM	10	12.0%
	HSU-EMIX	1	1.2%
Outcomes–HSU-U**	INS	10	12.0%
	VP	17	20.5%
	VH	6	7.2%
	EXAM	28	33.7%
	UPTAKE	18	21.7%
	DELAY	8	9.6%
	HSU-UMIX	3	3.6%

* Sample size for which associations between racism and HSU outcomes are reported.

** Numbers may not add to 100% (due to papers reporting multiple groups/exposures/outcomes).

*** Includes Major Discrimination, and instruments from the Detroit Area Study.

All papers were academic journal articles except one report. Papers were published between 1997 and 2015, with numbers showing a considerable increase over time; from just 2 (2.4%) papers published until 2000 and another 10 (12.0%) published between 2001 and 2005, to 30 (36.1%) published between 2006 and 2010, and 41 (49.4%) published between 2011 and 2015. The majority (95.2%) reported on research conducted in the U.S, while research from 4 (4.8%) other countries (Australia, The Netherlands, New Zealand and Sweden) was reported in one paper each. Most papers (96.4%) reported cross-sectional data, with the rest reporting longitudinal data. The sampling procedures used in most papers were non-representative (51.8%), and most of the remaining papers (47.0%) used representative sampling procedures. Most papers (57.8%) reported recruitment from settings not related to healthcare, with a minority (38.6%) reporting recruitment from healthcare settings (e.g., hospitals, clinics). Another 3 papers (3.6%) reported recruitment from both types of settings.

The total sample size of participants included in this review is 250,850 (range: 57–46,956). This number relates to participants included in analyses of racism and HSU from 70 studies. It was calculated after resolving potential duplication in multiple papers reporting the same study or data source. Where a study was reported in multiple papers only the largest N from these papers was used. Papers tended to report relatively large sample sizes: 42.2% reported sample sizes larger than 1,000 participants. Another 19.3% reported sample sizes ranging from 301 to 1,000 participants, while the remaining papers (38.5%) reported sample sizes of up to 300 participants.

Most papers (69.9%) did not focus on participants with any specific health or medical conditions, while 12.0% reported data from diabetes patients, 7.2% from HIV patients, and 2.4% each from patients with hypertension, heart conditions and osteoarthritis. Most papers included both men and women (73.5%), and most focused on adults only (85.5%). Over three quarters of papers (77.1%) included some or only African/Black American participants. Other commonly studied racial/ethnic groups included White/European Americans (48.2%), Hispanic/Latino/a Americans (41.0%), and Asian Americans (21.7%). Indigenous people (American and non-American) were included in 10.8% of papers.

The most commonly reported exposure instruments were the Everyday Discrimination Scale (EDS) (reported in 15.7% of papers), Experiences of Discrimination (EOD) (14.5%), Racism in Health Care Index (8.4%), and Discrimination in Medical Settings (DMS) (6.0%). Exposure measures using single item/s were reported in 39.8% of papers, and another 39.8% of papers reported exposure measures with 2–8 items. Exposures comprising 9 or more items were reported in 22.9% of papers. Most papers (83.1%) used measures of direct exposure to racism, and 16.9% of papers used measures of indirect, group exposure. Most papers used instruments that did not specify the exposure timeframe (55.4%), while 32.5% of papers used instruments that measured lifetime exposure to racism, and 20.5% of papers used instruments that used an exposure timeframe of 12-months. Exposures were interviewer-administered in 71.1% of papers, and self-administered by participants in 22.9% of papers. Most papers (59.0%) included exposure measures that focused on exposure to racism in healthcare settings (i.e., at least half their items were about healthcare), and 48.2% included exposure measures not focused on racism in healthcare settings. Due to several papers reporting multiple participant racial/ethnic groups, and different types of exposure and outcome measures, percentages of papers may not add to 100%.

Among HSU-E outcomes, satisfaction with health services and perceived quality of care (SAT) was reported in 18.1% of papers, followed by trust in healthcare system and professionals (TRUST; 14.5%), and communication and relationships with health service professionals (COM; 12.0%). Among HSU-U outcomes, the most frequently reported outcome was having had examinations, tests, screening and/or checks (EXAM), reported in 33.7% of papers. This was followed by having followed prescriptions, recommended treatments and behaviours, and having taken medications and vaccinations (UPTAKE; 21.7%), visits to health professionals (VP; 20.5%), having insurance and/or healthcare (INS; 12.0%), delaying/not getting healthcare (DELAY; 9.6%), and visits and admissions to hospitals and emergency departments (VH; 7.2%).

### Meta-analysis

The meta-analysis includes 59 papers that report data from 52 studies. It comprises 191 unadjusted associations between racism and HSU outcomes. Overall, the meta-analysis aspect of this study uses data from 167,063 participants.

**Fig 2:**
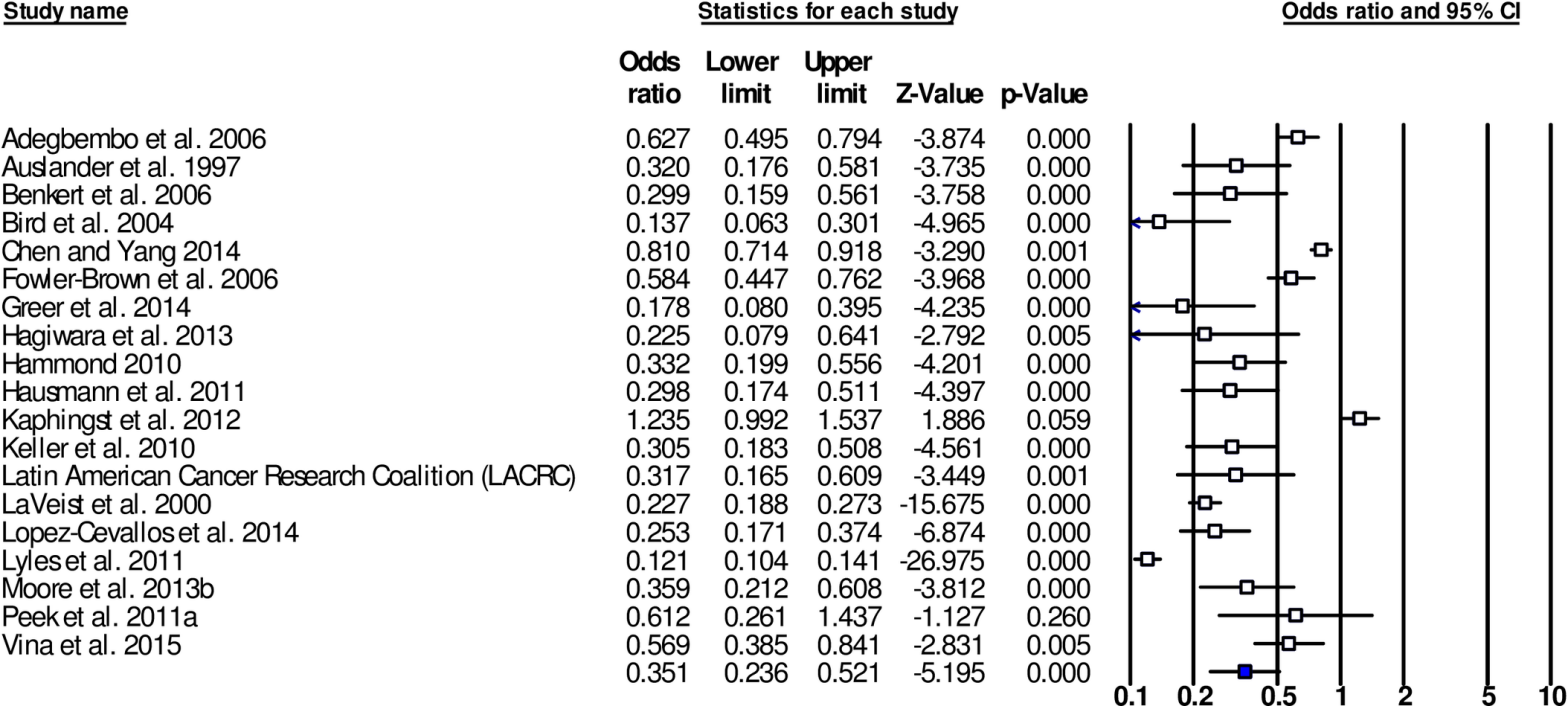
Forest plot of the effect sizes for associations between racism and health service utilisation-experiences (HSU-E) (k = 19).

**Fig 3:**
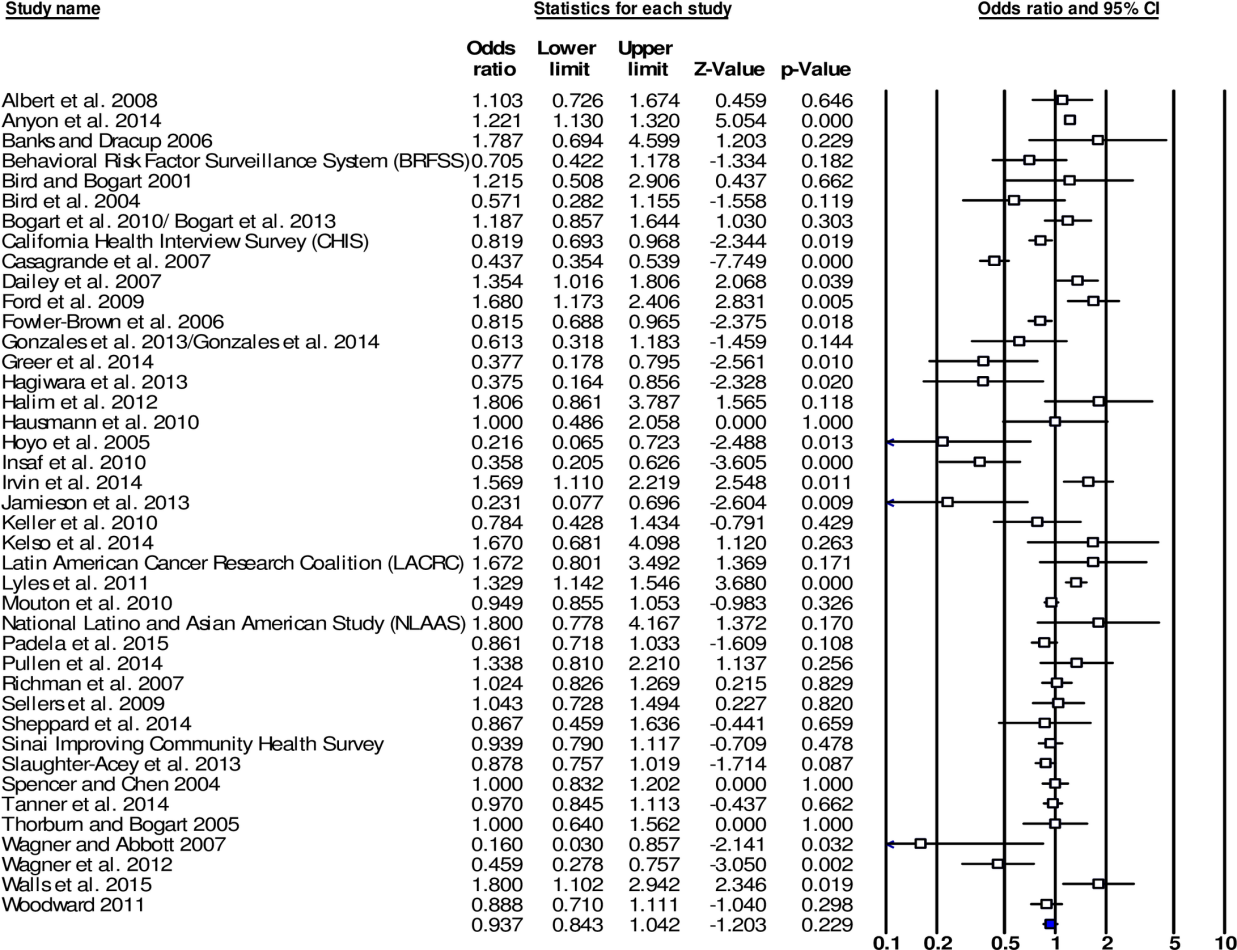
Forest plot of the effect sizes for associations between racism and health service utilisation-use (HSU-U) (k = 41).

Mean weighted effect sizes for the associations between racism and HSU outcomes are presented in Table 2. Forest plots are presented for the main outcome groups, HSU-E and HSU-U (see Figs 2 and 3). For all HSU-E outcomes, mean weighted effect sizes were significant and in the expected direction, indicating a negative association between racism and HSU-E outcomes. For the combined outcome group HSU-E the mean weighted effect size was OR = 0.351 (95% CI [0.236,0.521], k = 19). Effect sizes were similar for individual HSU-E outcome groups. The largest mean weighted effect size was for TRUST (OR = 0.312, 95% CI [0.165,0.589], k = 10), followed by COM (OR = 0.369, 95% CI [0.150,0.909], k = 7), and SAT (OR = 0.421, 95% CI [0.314,0.564], k = 9).

**Table 2:** Meta-analysis–weighted mean effect size association data for racism and HSU outcomes.

Outcome group	Outcome	OR	Lower CI	Upper CI	Z-value	p-Value	Number Studies k	Q-Value	df(Q)	P-value	I-squared
HSU-E	COM	0.369	0.150	0.909	-2.167	*0.030	7	311.992	6	0.000	98.077
	SAT	0.421	0.314	0.564	-5.801	*0.000	9	60.511	8	0.000	86.779
	TRUST	0.312	0.165	0.589	-3.587	*0.000	10	342.057	9	0.000	97.369
	HSU-E	0.351	0.236	0.521	-5.195	*0.000	19	540.172	18	0.000	96.668
HSU-U	EXAM	0.980	0.881	1.090	-0.365	0.715	17	40.921	16	0.001	60.900
	UPTAKE	0.700	0.541	0.907	-2.700	*0.007	12	28.724	11	0.003	61.704
	VP	1.091	0.930	1.279	1.067	0.286	13	39.753	12	0.000	69.814
	VH	1.375	0.905	2.089	1.491	0.136	4	7.730	3	0.052	61.192
	DELAY	0.430	0.357	0.519	-8.820	*0.000	3	0.534	2	0.766	0.000
	INS	0.886	0.561	1.399	-0.519	0.604	7	239.578	6	0.000	97.496
	HSU-U	0.937	0.843	1.042	-1.203	0.229	41	213.507	40	0.000	81.265

* p < 0.05.

The effect size for the combined outcome group HSU-U was non-significant (OR = 0.937, 95% CI [0.843,1.042], k = 41). Among individual outcome groups, significant effect sizes were recorded for DELAY (OR = 0.430, 95% CI [0.357,0.519], k = 3) and for UPTAKE (OR = 0.700, 95% CI [0.541,0.907], k = 12). For other individual HSU-U outcomes, effect sizes did not reach significance. This included INS (OR = 0.886, 95% CI [0.561,1.399], k = 7), EXAM (OR = 0.980, 95% CI [0.881,1.090], k = 17), VP (OR = 1.091, 95% CI [0.930,1.279], k = 13), and VH (OR = 1.375, 95% CI [0.905,2.089], k = 4).

Funnel plots were fairly symmetrical for all outcomes with the exception of SAT, where studies were concentrated in the lower left part and upper right part of the plot. Egger’s regression intercept was statistically significant for SAT, and non-significant for other outcomes. Using trim-and-fill procedures, 3 studies were imputed for SAT. Imputing resulted in a minor reduction in the effect size (adjusted from OR = 0.421 to OR = 0.516, 95% CI [0.395,0.676]), which remained significant, indicating that the impact of bias is likely to be trivial. Rosenthal’s failsafe N for the outcome UPTAKE was 47 studies, which with an average effect size of zero would render the effect non-significant. Since this value is smaller than the failsafe N criterion of 5*k* + 10, it indicates that the significant effect for UPTAKE may be an artifact of bias. For other significant results, failsafe N was well above this criterion.

### Moderation analysis

We examined whether associations between racism and HSU outcomes were moderated by participants having specific health/medical conditions and by the setting where racism was experienced (healthcare focused vs. non-healthcare focused). Analyses were run per outcome group, where at least two levels of the moderator each included 5 or more studies. Data were sufficient for examining the impact of having health/medical conditions on associations between racism and the outcome groups HSU-E and HSU-U. Having health/medical conditions significantly moderated the effect of racism on HSU-E. The effect size was stronger for studies focused on participants with specific health/medical conditions compared with studies of participants without specific conditions or where no specific conditions were reported (Q(1) = 4.13, p = 0.042). Having health/medical conditions did not significantly moderate the association between racism and HSU-U. Data were also sufficient for examining the impact of the setting where racism was experienced on associations between racism and the outcome group HSU-U, as well as the individual outcomes EXAM, UPTAKE and VP, yet setting did not significantly moderate the associations between racism and these outcomes.

### Vote-counting analysis

The review includes 9 papers (reporting data from 7 studies) that altogether contain 33 associations between racism and HSU outcomes that adjust for age, gender and race, often alongside additional covariates. Several papers reported multiple associations. For racism and HSU-E outcomes, 5 papers reported 12 associations, all of which showed a negative relationship between racism and HSU-E. Ten of these 12 associations (83.3%) were statistically significant. Three papers reported OR point estimates, which ranged between OR = 0.483 and OR = 0.746, and were significant. The main HSU-E outcome group was SAT, for which 7 out of 9 associations (77.8%) were significant and negative. COM and TRUST had 1 and 2 associations with racism, respectively, and all 3 associations were significant and negative.

In addition, 6 papers reported 21 adjusted associations between racism and HSU-U outcomes. Of these, 14 associations were negative (66.7%), and 7 positive. Two of these 21 associations were significant (and negative) (9.5%), while the remaining 19 associations, negative and positive, were non-significant. The 2 significant associations were between racism and DELAY, out of 3 associations for this outcome (66.7%). The remaining 18 associations, between racism and EXAM (13 associations), UPTAKE (2 associations) and VP (3 associations), were all non-significant.

## Discussion

We have undertaken what is, to our knowledge, the first systematic review and meta-analysis focused on the relationship between experiences of racism and health service utilisation. We found a total of 83 papers reporting 70 studies, of which 52 studies had sufficient data to be included in a meta-analysis. Attesting to the nascent state of this field, the first paper included in this review was published in 1997, while almost half of the papers were published between 2011–2015, and more than 85% from 2006–2015. Over 95% of papers were from the U.S., suggesting that findings may be of particular interest to scholars and policy makers focused on racism, healthcare and their interconnections in the U.S. at this time of profound social and legislative discussions concerning these areas.

The results of our meta-analysis indicate robust unadjusted associations between racism and HSU experiences. Those experiencing racism had approximately 2 to 3 times the odds of reporting reduced trust in healthcare systems and professionals, lower satisfaction with health services and perceived quality of care, and compromised communication and relationships with healthcare providers. Findings for use of health care (HSU-U) were mixed, and largely non-significant. Experiencing racism increased the odds of delayed care or unmet need, although this outcome was scarcely reported, and increased the odds of not adhering to recommended treatment uptake. However, there was no significant impact of experiencing racism on having health insurance, examinations or visits to health professionals or hospitals, and further analysis indicated that the significant effect for treatment adherence may be caused by publication bias. These findings were supported by vote-counting analyses adjusted for demographic covariates, particularly with regard to more commonly reported outcomes, such as the significant impact of racism on satisfaction with healthcare, and the non-significant association with examinations.

These results suggest that, despite being much more likely to have negative experiences in health services and from healthcare providers, patients experiencing racism are no less likely to access and utilise healthcare. Continued engagement may be a function of need for healthcare, despite compromised quality, as suggested by a moderation effect found in the meta-analysis in which the association between racism and HSU experiences (but not use) was stronger for those with health/medical conditions. This indicates that racism may be a particularly detrimental experience for those most in need of the healthcare system.

In terms of limitations in this growing area of study, less than 5% of studies were conducted outside the U.S., less than 4% utilised longitudinal data, and more than half used non-representative sampling methods. Exposure measures with 9 or more items were reported in less than a quarter of papers and more than half the instruments utilised did not specify an exposure timeframe. Building on a good mix of studies recruiting participants from, and measuring exposure to racism in, either healthcare or other settings, it would be useful for future studies to conduct more research with participants from both healthcare and non-healthcare settings to expand the 3.6% of papers that recruited participants from both types of settings. Although there was no moderation effect of discrimination setting found in our meta-analysis, given how few studies have examined this topic [41, 57, 58, 113], further research is warranted. A more comprehensive analysis of pathways between racism and healthcare, accounting for the multiple, complex roles of patients’ health remains an important future undertaking as well.

There are several limitations to this review. A major limitation concerns our focus on studies published in English, therefore constraining the extent to which our findings may be generalised beyond certain national and linguistic contexts. Another potential limitation of this study is the lack of assessment of studies’ quality. Using critical appraisal tools has been uncommon in meta-analyses of racism and health more generally and remains a challenge in assessing observational studies that use diverse methodologies and measures. By focusing on published studies, this study may reduce the likelihood of including lower quality studies. However, we acknowledge that there remains a possibility that differential study quality may impact the association between racism and healthcare utilisation. This review focuses on self-reported experiences of racism rather than on other types of racism. While, as we show, such experiences may be significant in their impact on healthcare experiences and service utilisation, we encourage future research to also review, synthesise and meta-analyse studies that define racism in other ways, including ecological, experimental and other measures of racism where racism is defined by the researcher.

This systematic review and meta-analysis demonstrates a need for more longitudinal studies using probability-based sampling approaches that comprehensively measure both exposure to racism and a range of relevant health service utilisation outcomes over aetiologically-relevant timeframes. They also signal the dearth of research conducted outside the U.S. and call for additional exploration of the applicability of current findings across various national contexts and diverse healthcare systems. This research also justifies renewed efforts to reduce racism in healthcare settings, targeting both bias among providers [119, 120] and institutional efforts to improve the cultural competency of healthcare systems [121–124].

## Supporting information

S1 Appendix Search strategy for MEDLINE (adapted for other databases as needed). (PDF)

S1 Table PRISMA 2009 checklist. (PDF)
