# Supplementary material for: Racism and health service utilisation: A systematic review and meta-analysis
Source: PLoS One. 2017 Dec 18;12(12):e0189900. doi: 10.1371/journal.pone.0189900 (PMC5734775; doi:10.1371/journal.pone.0189900)
Supplement: S1 Appendix — (PDF) [file pone.0189900.s001.pdf]

S1 Appendix. Search Strategy for MEDLINE (adapted for other databases as needed)

Search 1 terms:

1 exp Prejudice/ or "racial discrimination".mp. or racism.mp. (21527)

2 health services/ or "health care quality, access, and evaluation"/ (17306)

3 professional-patient relations/ or trust/ (23223)

4 Patient Satisfaction/ (49180)

5 Physician-Patient Relations/ (54173)

6 Health Behavior/ (26321)

7 health behavior/ or patient compliance/ or medication adherence/ (69847)

8 2 or 3 or 4 or 5 or 6 or 7 (201717)

9 1 and 8 (1332)

10 from 9 keep 7,13,30,39,45,88,95,115,136,141,143,152,183,189,256,260,279,281,402,409,411,428, 444,467,481,499,510,526,539,546,552,565,588,634,663,723,811,862,1001 (39)

Search 2 terms (Paradies et al., 2015):

1 birth* (ts)

2 gestation* (ts)

3 health* (ts)

4 well-being (ts)

5 wellbeing (ts)

6 disease* (ts)

7 illness* (ts)

8 BMI (ts)

9 “body mass index”(ts)

10 WHR (ts)

11 “waist hip ratio (ts)

12 anthropometric* (ts)

13 “blood pressure” (ts)

14 hypertension (ts)

15 cardiovascular (ts)

16 overweight (ts)

17 obes* (ts)

18 depressi* (ts)

19 anxi* (ts)

20 distress (ts)

21 stress (ts)

22 suicid* (ts)

23 sleep* (ts)

24 (social* OR behav* OR emotio* OR develop* OR psych*) (AB) AND (difficul* OR problem* OR delay* OR adjust*) (ts)

25 self-esteem (ts)

26 “self esteem” (ts)

27 “life satisfaction” (ts B)

28 “quality of life” (ts B)

29 resilien* (ts)

30 alcohol (ts)

31 tobacco (ts)

32 smok* (ts)

33 “substance use” (ts)

34 drug* (ts)

35 health (ts) AND (care OR service* OR clinic*) (ts)

36 Diseases (MESH)

37 Psychiatry (MESH)

38 Psychology (MESH)

39 1- 38 (OR)

40 ts=(discrim* OR bias OR prejud* OR hostil* OR harass* OR bully* OR “unfair treat*” OR oppress*) OR mh=prejudice

41 ts=(rac* OR ethnic* OR cultur* OR religio* OR migra* OR immigra* OR refugee*) OR mh=ethnic groups OR mh= minority groups

42 ts=(longit* OR cohort* OR trial* OR “follow up” OR prospective OR retrospective OR “cross section*” OR cross-section* OR intervention* or quantitative or survey* OR “case-control” or “case control” or “randomised control* trial*” or “randomized control* trial*” or ”before and after” or “interrupted time series” or questionnaire* or registr* OR evaluat* or audit*) OR mh=Longitudinal Studies OR mh=Epidemiologic Research Design OR mh=Epidemiologic Study Characteristics as Topic or mh=registries

43 39 AND 40 AND 41 AND 42
